# Academy of Stomatology

**Published:** 1895-04

**Authors:** George D. B. Darby

**Affiliations:** Secretary


### ACADEMY OF STOMATOLOGY.

A REGULAR meeting of the Academy of Stomatology was held at the rooms of the Academy, 1731 Chestnut Street, Philadelphia, February 12, 1895, the President, Dr. Louis Jack, in the chair.

After the transaction of routine business, Dr. Edwin T. Darby read a paper upon "Gouty Pericementitis."

(For Dr. Darby's paper, see page 197.)

### DISCUSSION.

*Dr. M. L. Rhein.*—Mr. President and Fellows of the Academy,—I have had much pleasure in listening to the paper read by Professor Darby, and am sorry he was not present during the summer at the discussion on this subject, when a similar train of cases was presented before the American Dental Association at Old Point Comfort by Dr. Crouse, of Chicago. Dr. Crouse very graphically described the cases and dwelt at length upon the fact that these molars were attacked individually,—precisely in the same way that has been illustrated in the paper this evening,—that nothing was left to him but to extract the teeth, and that he had found this condition of affairs on the end of the root. After he had taken his seat one of the members of the Association from New York took exception to Dr. Crouse's statements, inferring that he was not competent to observe properly. I suppose what I say may recall it to the memory of many of the gentlemen who were present at that meeting. I personally felt like verifying the accuracy of Dr. Crouse's statements that such pathological conditions did exist. That they have not been more frequently reported is unquestionably due to the difficulty in diagnosis and the common method in which we relegate at the present time the extraction of teeth to the specialist in extraction, when we, as a rule, do not see the teeth that are removed by them.

The point that interested me most was the question of the proper nomenclature of this subject. I presented a hastily-written article on the etiological classification of pyorrhœa alveolaris at the last meeting of the American Dental Association, which was published in a recent number of the *Dental Cosmos*, and in that article I claimed that we had no right to depart from the beaten and accustomed track of medical science generally in classifying a disease and subdividing the classifications according to clinical appearances, but ought to follow what has held sway in all the departments of medicine, and classify strictly according to its etiology. Now, my experience with all pericemental inflammations has been that they are as prolix as the diseases with which we are liable to meet, and that the clinical results that follow vary more or less in their symptoms and appearances according to that cause. That we may meet with some much more frequently than others, and that some cases may be more common in certain sections and in certain classes of people, seems a very reasonable conclusion. In that article I preferred to retain for all forms of pathological condition, where there was or may have been a flow or an exudation of pus from the gingival border, the name by which it was most commonly known,—that is, pyorrhœa alveolaris,—and then subclassify it by adding as an adjective of any case the etiological cause of such particular case. That is to say, the cases as presented this evening, after we had obtained a condition of pyorrhœa alveolaris, after there had been a pocket formed with suppuration, could be called pyorrhœa rheumatica or arthritic rheumatica, according to the wish of the party describing the same.

Now, I also recognize the fact that the condition of affairs existing in these cases that seem to be rather rare to us at the present time, whether they are as rare as they seem, is an uncertain thing at present. But it seems to me that such cases where suppuration does not exist,—where we simply have a deposit near the ends of the root that acts as an irritant,—it seems to me that we could not call that a condition of pyorrhœa. We do not have that condition present, but we do have present a condition of pericementitis, which is very liable to develop into a condition of pyorrhœa alveolaris. The moment we refuse to adopt a common name and then subdivide it according to its etiology, we are bound to be met continually by the differences of opinion, by different men from different parts of the world, not understanding the particular case under description. Last summer I spent some time in visiting the medical wards of one of our large hospitals in New York City, and examined the mouths of the patients there, from the incipient stages of typhoid fever to the convalescent stages, and also a number of cases of chronic invalidism that were in various stages of treatment. In none of these cases was there such a condition as a healthy mucous membrane. The point I wish to illustrate is, that wherever we get a nutritive starvation, there is not sufficient to thoroughly supply the human economy with pabulum for the amount of wear and tear of the daily cell-life; the part of the tissue that is soonest to feel that paucity of newly-made blood-corpuscles for enriching the supply would naturally be the remotest portions of anatomy,—that is, that portion that is reached by the remotest capillaries of any portion of the circulatory system. And in that part of the body we certainly are aware that the capillary system supplying the peridental membrane and the gingival border along the roots are the remotest portions, and these conditions seem to be theoretically the only outcome right there of malnutrition.

In all these typhoid-fever cases there was more or less a condition of pyorrhœa present around the gums. In some it was very marked. I call particular attention to this point to emphasize the necessity in my mind for this sort of classification, for if my observations are correct, we are liable to a pyorrhœal condition in any form of disease that will subject the nutritive action to a lowered standard. This is a point upon which I would like to bring out a reply from the writer of the paper, principally because at the commencement of his article he devotes considerable attention to the admirable papers of Dr. Peirce on this subject, and it is merely a question whether it does not draw the line too closely around a very large and much wider circle than the one form of gouty disease can possibly do.

*Dr. M. H. Cryer.*—In Dr. Darby's paper he described an apparent abscess opening on an upper molar tooth. Since our last meeting a patient, whom I have had some eighteen years, called, complaining of a second bicuspid. On examining it over the root I found an abscess, and on tapping the tooth it responded as though there were pericemental inflammation, and indicated a devitalized tooth.

In drilling it, rather carefully at first, and no response coming,—I perhaps ran the engine a little fast,—the patient made a sudden jump, and, hastily removing the instrument, I found blood. On examining the tooth I found a vital pulp. The abscess was not immediately over the point, but very close. The first molar had previously been devitalized, and, finding this condition, I removed the filling and dressings, but found them in perfect condition. After exploring the roots carefully, I came to the conclusion that the molar tooth had nothing to do with the abscess. Knowing that Drs. Kirk, Darby, McQuillen, and Huey would meet at Dr. Darby's office yesterday, I asked the patient to call around. Dr. Darby kindly removed the dressings in each tooth, and I believe that these gentlemen support my opinion that the abscess is at or near the apex of the bicuspid tooth, and that the bicuspid tooth, or something associated with that tooth, is the cause. On taking out the dressing yesterday morning there was a very large flow of blood, although the tooth had been comfortable for several days. This blood could not come from any other than the inflamed condition down through the tooth, although the pulp is vital.

I would like further to speak—for it is bearing on the subject that we have before us—of an abscess opening on the face, apparently coming from a diseased tooth. A member of this society a year ago sent me a patient with a swelling on the lower jaw just opposite the second molar. The first molar, a devitalized tooth and filled, did not respond to any percussion, and appeared to be in a good healthy condition. I sent the patient back to his dentist, with the suggestion that it should be closely watched; if the swelling should increase or the tooth become painful, the tooth should either be opened—that is, the filling removed and treated—or extracted. If I remember aright, the patient did not go to his dentist for over three months. He finally came back to me with an abscess about ready to point. I lanced it and passed the probe into the opening in the direction of the first molar. There was not any time to waste, so the tooth was extracted, and then with very little trouble the probe was passed from the outside into the alveolus and out through the opening into the mouth. I found the edge of the bone somewhat diseased, so I bored it away with the engine and packed the parts with iodoform gauze, passing a part of it out through the opening; the parts healed almost immediately, and within a week's time the patient was discharged.

I have taken the liberty of bringing a patient here to-night who had trouble with a second molar, very sensitive, all the indications of pericementitis. The tooth became so painful it was extracted last September. It was swollen on the outside along the lower portion of the bone, increasing in size, and, becoming more painful, a physician lanced it, getting a small amount of pus. This happened last September, and passed into my hands in the latter part of December. In passing a probe through the opening it went to the bone; then, by curving it, I passed it under the bone, and on placing the finger could recognize the point of the probe under the mylo-hyoid muscle. The parts were syringed with a peroxide of hydrogen solution and showed some evidence of pus. Of course the serum will cause the frothing or bubbling, but from certain experience it was evident that there was also pus in this pocket. The part was opened slightly, iodoform gauze was packed tightly down to the bottom, passing a portion of it out, and leaving it hanging out of the opening. This treatment was carried on until it was impossible to repack it, and it had all the appearance of getting well, but in a few days the pus began flowing again. About three weeks ago I had him before a clinic, and examined by one of our celebrated surgeons and one or two others. We all, however, failed, I think, to make a diagnosis. At this time the parts were opened considerably and a probe passed in the end, and the treatment was carried on as before with the hope that this time it would cure, but it failed. The next day after it was opened part of the fluid taken from the opening was examined under the microscope, with no result. There has not been enough to make a chemical analysis to see if it is from the salivary glands, though it is possible to have saliva flowing in this region. The serum has been put under cultivation to see if any tubercular bacteria would develop, but such has not been shown. On a second treatment those who had not seen the wound before were satisfied it was going to close, but that was the supposition of all who had seen it for the first. It finally did close, but on the second day there was again this discharge. The family history is good, there being no specific taint. I should like our friend from New York and others here to see the patient and see if he can find a diagnosis. The diseases of the pericementum and the inferior maxillary are so close that I think it really borders on it. But at Dr. Darby's suggestion the discussion will go on and we will see the case afterwards.

*Dr. Howard Roberts.*—Dr. Darby makes the remark that he seldom sees pyorrhœa in a patient under thirty. I wish to say that I have a patient, who is not over twenty-six, that I think has the most stubborn case of pyorrhœa I ever saw. And it started two or three years ago. The two bicuspids—one on either side—are very much affected, one of them is very loose. There is not much discharge of pus, and very little inflammation. I should like to know a treatment for it that will cure. I have now put her on tartrate of lithia to see if that will have any effect.

*Dr. E. C. Kirk.*—I would like to speak a few words about these abscesses that form in relation to the acute condition. I reported at the last meeting of the Academy some abscesses of this sort that I had observed in the mouths of patients, and also that I had very closely observed in my own mouth. Since the last meeting I have been watching these cases, and they have developed one or two features which seem to me to be interesting. They are interesting as showing what may be called the mechanical factors which determine to a certain extent the character of pyorrhœa. I have seen two cases where a pericemental inflammation resulted in the formation of a discharge through the gum tissue. I would not characterize it as pus, and I question whether the condition would be characterized as an abscess. However, the inflammation resulted in a formation of a discharge on the gum surface. It did not break through between the alveolus and the gum tissue and discharge at the gingival margin, but directly out through the tissue upon the gum. And that inflammatory condition healed almost at once.

I think we have all seen cases of alveolo-dental abscess terminate in the same way,—those which start and heal up without the formation of a chronic fistula. On the other hand, I have observed cases of acute abscess, such as Dr. Darby has described, where the fistulous tract was established between the soft tissue and the tooth-root,—that is to say, the discharge took place on the gingival margin and a probe could be passed down almost to the apex of the root. Now, this case refused to heal up, and has continued to present an appearance of one of the forms of pyorrhœa alveolaris.

There is another feature which seems to me related to this point,—the character of the tissue as a modifying factor in which the inflammation is expressed. Dr. Darby has drawn attention especially to the pericemental membrane as the seat of this inflammatory condition, and draws an analogy between the gouty inflammations of membranes and this and other ligaments. In Dr. Black's book upon the histology of the pericemental membrane, and more recently, he has called attention to the existence of true epithelial glandular structures that are certainly found in that tract, and in a later publication—if I mistake not, it occurred in the *Dental Review* in criticism of Dr. Peirce's paper—he stated positively and without qualification that this glandular structure is situated in what he calls the gingival space, and is the seat of pyorrhœa alveolaris. Now, when Dr. Black makes a statement in that manner he certainly believes that it is correct, and he has made it in the light of careful investigations; therefore, for our purpose we can accept that it is true, and I call attention to it because it has a bearing on certain cases of pyorrhœa.

About a year ago a patient presented herself to me with her teeth all perfectly sound. Less than six months after that she called for an examination, and I found erosion taking place, slight, comparatively, and yet it was rapid, having all been established in the interval between her two visits, and seemed to be of such rapid progress that I decided that it was best to fill these cavities. I remember having some difficulty in trying to decide whether I should or should not fill with gold. I saw her the next six months after that, and I was horrified to find that these fillings which I had inserted were standing up like islands in the midst of the sea, the tooth substance melting away from around them, with the enamel for a considerable extent beyond them rather chalky-looking as if it had been immersed in a dilute acid. I then began to note the general condition of the patient, and I found that she was exceedingly plethoric, full habit, florid, looking as though she might at any time be stricken with an attack of apoplexy, and the gums presented the same turgid engorged appearance. I called her attention to these points, and suggested that she consult a physician. As she had no family physician, I sent her to a medical man in whose judgment I had confidence, and asked him to give me a report of her physical condition. He wrote me a letter within a few days stating that she was a marked and typical case of uric acid diathesis, and that accounted for her systemic manifestations. On her next visit I spoke to her about her condition, and she said that she had been in Europe at various spas for treatment, had been in Carlsbad, and under treatment for it before, but she had developed either there or at some place in her continental tour a goitre, for which she was under treatment here and was taking thyroid extract. Within a week or ten days afterwards she came to me and said that some one had told her that thyroid extract is little less than a solution of uric acid. Her physician had insisted that she should take it to cure the goitre, but, as she said to me, Why should I take this solution of uric acid if it is going to have this effect on me? I confessed it was a reasonable proposition, and, on her own responsibility, she stopped taking thyroid extract and went under a treatment with lithium bitartrate. In the course of two or three months her goitre had left her, her thyroid gland had become reduced to nearly normal, the condition of her mouth was entirely changed, she had lost that full, engorged, plethoric, hyperæmic appearance, and she felt like another person. She has been keeping up a modified gout regimen, and there has been certainly an arrest in the progress of her erosion.

It seemed to be a very interesting observation to find the effect of uric acid upon the glandular structure of the thyroid as well as of the labial mucous glands, which we now know to be the cause of the erosion; and that these two causes, in connection with the possible glandular involvement of pyorrhœa, are produced by uric acid condition are points which seem to me to be worthy of consideration.

*Dr. Rhein.*—There was a word that I wanted to add that escaped me at the time in reference to the cases Dr. Darby mentioned, and that is the treatment of those cases. I have had a little success in some of those cases by following a strictly surgical line of treatment,—practically the same method that we would pursue if we had a pocket at the gingival border. Invariably I have devitalized these pulps. It is my opinion that the pulps in such teeth are better devitalized than left to perhaps a worse fate.

*Dr. Kirk.*—Why would you destroy them?

*Dr. Rhein.*—There are a number of possibilities. There is a possibility of uric acid deposits in the pulp itself. To my mind, if it is possible to obtain these deposits around the periphery of the tooth which receives its supply from the same vessel that simply divides up around the end of the root, it is just as possible to get some form that we speak of in a rough manner, a pulp-stone in the pulp itself, and produce some of those terrible neuralgic conditions that we meet with of that cause. At any rate, it has become a common practice with me to immediately devitalize these teeth.

Secondly, it is my custom to remove a portion of that root, if not the entire root that is involved. In a superior molar I have removed one of three roots so frequently, and the results have been so gratifying to me, that it is an operation that I look on with a great deal of favor. There is no other tooth in the mouth that permits of such an operation with such gratifying results as a superior molar; and I want simply to point out this method of treatment for the salvation of teeth that are attacked in this manner, especially if only one of the roots be involved; and while it is a little foreign to the subject, in a number of cases of pyorrhœa alveolaris where the surrounding teeth are liable to infection from the pericemental root, which we find in older people entirely denuded, the superior molars do good service.

*Dr. H. H. Burchard.*—What Dr. Rhein speaks of as advisable to do, nature does herself in a great many cases. In a paper read before the County Medical Society some time ago, I assumed that in patients the subjects of the gouty diathesis the morbid conditions would not break out with volcanic force. It would be existent over a long period and the acute outbreak was the culmination. In all probability the excessive uric acid condition exists for years before there is an acute manifestation.

Cell activity emphasizes itself in several ways. Probably the first form is in higher organization,—that is, in many parts formed material becomes harder, and so on, acting upon the odontoblasts, the peripheral cells of the pulp, they are stimulated to a greater activity, and the dentine becomes more dense. Continue the irritation long enough in the proper degree, and there will result self-obliteration of the pulp. This is an early stage of disease-action, the stimulative. The next stage in severity will be the irritative. One of the characteristics of this is altered secretion. In a discussion of a most excellent paper of Dr. Darby's, read some few years ago, Dr. Kirk advanced the opinion that the acid, the cause of gouty erosion, was the product of the mucous glands about the part. Dr. Brubaker has recently written upon this subject also. The irritated secretory apparatus produces altered products, acid in reaction, and these act as decalcifying agents. The third stage, following the stimulative and the irritative, is the necrotic. At this point, I really do not see, if the loss of a tooth occur through—I will not call it pyorrhea alveolaris, as this term merely names a symptom, not a disease—a progressive degeneration of the pericementum, and due to the presence of uric acid in the circulating fluids, why there should be deposits, and why pus-formation, granting that pus always represents micro-organisms, which I do not believe. If this loss of articulative tissue be a purely gouty affection, the pathology of this type of pericementitis should accord with that of gout in any other articulation. Ebstein states that necrosis precedes deposits in gout,—that is, necrosis of the tissues precedes the deposits of urates in an area. Preceding the necrosis there is an inflammation of the blood-vessels. One of the effects of this is swelling of the tunica media. In small vessels this may be sufficient to occlude the lumen, and there will be death of elements dependent upon this vessel. I forget what pathologist states that necrotic tissues in this disorder particularly have an acid reaction. The urates insoluble in acids are precipitated in this area, a chemical reaction purely. I believe in and have found the association frequently of a gouty pericemental degeneration, which begins, not as the preceding, near the apex, but just beneath the margin of the gum. I am not yet convinced of the presence of epithelial tissue in the pericementum; but even though mucous glands are the only ones about the gingivæ, their altered secretion may serve to cause deposits from the saliva. The disease progresses then as any inflammatory degeneration. These may be gouty cases, and yet may be curable by keeping them free from deposits.

*Dr. S. H. Guilford.*—Pyorrhœa alveolaris is a disease that expresses itself in various ways. I remember the case that Dr. Darby referred to, the first case he mentioned, because we were then neighbors; calling upon him the very evening of the day that this case occurred, he asked me if I had ever seen a case of abscess where the pulp was alive? We thought it very wonderful in those days, and yet we would not think so to-day.

Shortly after that there came to me at my office a negro policeman who was suffering with one of his upper molar teeth. The tooth was perfectly loose and the gum very much inflamed. I extracted the tooth. He was one of the finest specimens of physical development I had ever seen, and yet was subject to this peculiar condition. I kept the tooth very many years, and I think I may yet have it somewhere, because of the extent and character of the deposit on the root.

Not long ago a lady came to this city from Pittsburg who had been under treatment there by a dentist, and as she was coming here she was referred to me. She had a very beautiful set of teeth, but around the superior left lateral incisor the gum was very much inflamed and it was receding from the neck of the tooth. It gave her a great deal of trouble, and she wanted some relief. I examined the case for some time very carefully indeed, and saw it a number of times, extending over a period of several months. The characteristics were entirely different; there was pain, inflammation, and erosion; there was loosening of the pericementum, but no deposit at all upon the root that I could possibly find. I took a probe time and again and passed it up almost to the apex of the root, and with all the care that I could exercise I could not discover any evidence of the deposits. There was no flow of pus; in fact, no discharge of any kind. I advised the removal of the lateral incisors, but she decided not to have it done.

I met the physician who had charge of her afterwards. He asked me what success I had had, and I said, none. I was able to learn that she was troubled with inflammation of the ovaries, and the physician had been trying to help her in various ways, but was not able to do so. He advised a surgical removal of the ovaries, but she was not willing to have it done.

In her case, I think, it was nothing but a reflex manifestation or exhibition of the disordered condition of her ovaries; nothing more than an abnormal condition of one part showing itself very markedly in the teeth.

I mention it particularly because there was an entire absence of any deposit on the teeth, or of pus, serum, or any discharge whatever.

*Dr. A. W. Deane.*—At one of the previous meetings I reported a case that has come under my care, of two of the lower incisors, the pocket having formed beneath the gum margin and worked itself out to the surface. As I said at that time, I placed her under Dr. Peirce's treatment and gave her unlimited quantities of lithia, and the case has responded very favorably; but her physician steps in now and says that she must not use lithia; there being a trace of consumption in the family, he is afraid that she would develop something, he did not know what, from the use of lithia. What I want to know is whether she shall change her doctor or change her dentist. The pus has entirely stopped in its flow, and her general health seems better. I have never before heard of any advice of that kind coming from a medical source.

As to what our New York friend suggests as to devitalizing the teeth, in a paper by Dr. D. D. Smith, recently read before the Odontological Society, he advocates devitalizing the pulp in such cases.

*Dr. Burchard.*—Judging from what has been said about the ages of these patients, it would seem to me that too much attention is paid to that detail in regard to diseases. There is no reason why a disease that breaks out at thirty should not appear at the age of twelve or thirteen or fourteen.

For instance, encephaloma has been found in a child eighteen months old, and it is well known that it never makes its appearance except in adults. These things simply teach that there are exceptions to the general rule, and general rules in medicine are of but little value.

*Dr. Jack.*—The chair has three cases bearing upon this subject, which are each of different character, that he would like to state.

CASE I.—Mr. W., left upper central. Tumefaction and abscess of gum. Tooth was alive. A patient nearly fifty years of age, of gouty diathesis, subject to neuralgia and severe headaches. The attacks have been frequent and maddening. Habit full, complexion florid. Applied with swelling of the gum over left central and lateral. Teeth but little sore. Margins of gum red, with very thin scale of black tartar beneath them. At the central portion, over the middle of the central incisor root, there appeared a small fluctuating spot which when opened discharged a couple of drops of pus. The bottom of the excavation did not apparently reach the peridontium; the cavity of the abscess was defined, globular in form at the bottom, and shining. After removing the salivary calculus the parts became restored to health. The pulp in each of the teeth was vital and their color normal. Since then a deposit of the same character of calculus has appeared, accompanied by tumefaction of margins of the gum, but without the appearance of pus.

CASE II.—Mrs. N. Right upper central incisor had repeatedly appeared with tumefaction of gum over the tooth extending from near the margin to the connection with the lip. The appearances presented the same character as the early stages of alveolar abscess, but without the usual characteristic soreness of the tooth to pressure or percussion. On testing found it sensitive to cold. With each attack the tooth appeared to lose its healthy tinge. After many years the tooth lost its temperature sense, when from the changes of color, some soreness occurring in connection with the tumefaction, it was decided to remove a cervical filling and drill into the pulp-chamber. I may state that the period during which this tooth was under observation was twenty years, and these attacks were not infrequent. In a moment sensibility was manifested. A year or more afterwards the patient asked that the pulp be destroyed to enable the tooth to be bleached. This was done, the pulp being found fully vital. The peculiarity of the case is this, that in many instances these attacks immediately followed the taking of wine, the patient averring that she could not use wine without an attack supervening. The patient was evidently of gouty diathesis, which at the first—which was twenty years ago—was not indicated except by the appearance of considerable erosion of the teeth, but of late years this is markedly her condition, since the joints of the fingers show unmistakably the tendencies in this direction.

I present these two cases for what light they may throw upon the discussion of the last meeting upon the subject of the influence of gouty diathesis upon the tissues contiguous to the teeth.

Case III.—Mrs. R. A further case of somewhat different character, but indicative of the influence of this gouty diathesis upon the peridontal membrane, may be related in this connection.

A patient came complaining of reflected pain in the teeth of both sides of the face. She had several capped pulps, and while there appeared no reflection of the pain to the ear, which is an almost invariable accompaniment of pulp irritation, I made the usual external application over them without any benefit. On her second return the complaint was of soreness of all the teeth, which prevented her from using them at the table. An examination revealed the fact of the existence of peridontal irritation of nearly all the teeth. I advised her to apply to her physician for treatment for apparent lithæmia. He confirmed this diagnosis. The patient undoubtedly possessed a strong inherent tendency to gouty disturbances, and had been treated for other gouty manifestations. Moreover, the inheritance in her case is undoubted.

*Dr. William H. Trueman.*—This is not a subject that I have made a special study, for my inclination runs in another direction. I think Dr. Darby in his paper stated that these diseased conditions are rather on the increase. I think that the reason is that these conditions belong to middle life and beyond, and at the present time more of our patients have reached middle life than we had twenty years ago.

I suggest to those who are especially interested in this subject that it may save a lot of threshing of old straw if they were to take the literature of this subject and read what has already been published. They will find plenty of books on it. They think that dentistry is but a few years old. In 1858 there was sold in London a house that had been occupied as a dental office for nearly two hundred years. It had to go to make room for improvements. Dentistry is not a modern science. I hold in my hand a book upon the subject we are discussing—"Morbid Conditions of the Gums"—published in 1722. These subjects have been considered by students of years gone by. You will find in the literature of the subject quite large treatises—text-books—which refer to the subjects which we take up as being new discoveries.

*Dr. J. A. Woodward.*—A young gentleman, twenty-two years old, of full habit, a high liver, and of moderately heavy circulation, developed an excessively sore mouth; that is, last spring he went abroad, and while on the Continent he returned to London with this sore mouth. He fell there into the hands of a physician, and then a dentist; the latter did best. He came back to me in October with one of the worst inflamed mouths I ever saw. On examining the teeth carefully there was no tartar, there was no pus. The tooth was tender to pressure, and to heat and cold. I of course tried to get the mouth into some sort of condition as to cleanliness. He could not bear any brush. The softest brush I could get could not be borne, so I had to resort to mouth-washes and to antiseptic treatment. In despair I turned him over to one of our prominent men here, and that gentleman started with the same idea that I had,—that it came from the stomach, and was from smoking.

He got nothing out of that. I examined the teeth on Saturday. I found the mouth very much improved, the swelling had disappeared, the gum had returned to its natural color, the teeth were still loose, but the man was able to brush them. I wrote a note to the gentleman, and this is his reply: Mr. R.'s mouth offered a very obscure problem, and I changed nothing for him until I made up my mind that the condition was gouty.

On motion the subject was passed.

George D. B. Darby,
*Secretary.*

